# Supplementary material for: Multi-criteria group shilling attacks
Source: PLoS One. 2025 Dec 11;20(12):e0338319. doi: 10.1371/journal.pone.0338319 (PMC12774353; doi:10.1371/journal.pone.0338319)
Supplement: Attachment — Submitted filename: ResponsesToReviewers.pdf [file pone.0338319.s001.pdf]

Response to the Reviewer's Comments

[Multi-Criteria Group Shilling Attacks]

Tuğba Türkoğlu Kaya

October 26, 2025

Dear Editor and the Reviewers,

Thank you very much for your email and for giving us the opportunity to revise our manuscript submitted to *PLOS ONE*. We sincerely appreciate the valuable and constructive comments provided by the reviewers and the academic editor.
We will carefully address all the points raised during the review process and prepare a detailed **Response to Reviewers** document, along with a revised version of the manuscript including tracked changes. In addition, all modifications made in response to the reviewers' comments have been clearly highlighted in red within the revised manuscript to facilitate the review process.
We aim to submit the revised version before the given deadline (December 6, 2025).
Once again, we would like to express our gratitude for your consideration and for giving us this opportunity to improve our work.
Best regards

COMMENTS BY THE REVIEWERS

Comments by Reviewer #1

This paper presents a valuable and timely study on the robustness of multi-criteria group recommender systems under coordinated adversarial manipulation, known as group shilling attacks. It introduces a novel adaptation of the GSAGen model to multi-criteria settings (MC-GSA) and provides an extensive experimental evaluation on Yahoo! Movies datasets (YM10 and YM20). The topic is original and relevant for the recommender systems community, addressing a clear research gap. The manuscript is well structured, and the methodology is sound. However, several sections could benefit from clarification, deeper discussion, and minor language and presentation improvements before publication.

1) The abstract is informative but should briefly state the main quantitative findings (e.g., percentage impact or robustness level) to better convey the results.

Response: We appreciate the reviewer's valuable suggestion. In accordance with this comment, we have revised the abstract to include the main quantitative outcomes obtained from the experimental results. Specifically, the revised abstract now highlights that the proposed multi-criteria recommender system achieved up to 12% AvgHR improvement on the YM20 dataset and 15% on the YM10 dataset, demonstrating strong robustness against shilling attacks. Furthermore, we explicitly state that the MUP-NNZ item selection strategy provided the most stable performance under attack scenarios. These additions make the abstract more informative and quantitatively descriptive, as recommended.

2) Clarify the novelty claim: while this is presented as the "first comprehensive robustness analy-

sis," include a brief comparative statement positioning this work against recent studies (2022–2024) in multi-criteria RS or attack detection.

Response: We appreciate the reviewer's insightful comment regarding the novelty clarification. In response, we have revised the last paragraph of the Introduction to more clearly define the contribution and to position our study with respect to recent works (2022–2024) in multi-criteria recommender systems and attack detection. Specifically, we now emphasize that while several recent studies (e.g., Kaya and Kaleli, 2022; Turkoglu et al., 2023) have investigated individual-level attack detection in multi-criteria recommender systems, none have conducted a systematic robustness analysis under group shilling attacks. We explicitly clarify that our work constitutes the first comprehensive robustness evaluation of multi-criteria group recommender systems (MC-GRS) against coordinated (group-based) adversarial behaviors.

The revised paragraph (in the Introduction section) now reads as follows:

"Despite the significance of this threat, the existing body of research reveals a critical gap... [line 50-55]."

3) Some sentences in the introduction are repetitive (lines 5–25); they could be condensed to improve flow.

Response: Thank you for this suggestion. The introduction section has been carefully revised to remove repetitive sentences and improve the logical flow. The discussion of user data collection, feedback importance, and multi-criteria examples has been condensed into a more concise and coherent narrative while maintaining the key contextual details.

4) The literature review is comprehensive but should reference a few recent studies (post-2020) on adversarial or poisoning attacks in recommender systems beyond shilling (e.g., GNN-based adversarial robustness).

Response: We sincerely thank the reviewer for this valuable suggestion. In accordance with the comment, the *Related Work* section has been carefully revised to include recent studies (post-2020) addressing adversarial and poisoning attacks in recommender systems, particularly those employing Graph Neural Networks (GNNs) and robustness-oriented frameworks. Specifically, the updated section now references the works of Wang et al. (2024) on distributionally robust GNN-based recommendation models and Boratto et al. (2023, 2024) on robustness–fairness trade-offs under edge-level perturbations. These additions broaden the contextual scope of our study, aligning it with recent advances in adversarial robustness research while clarifying how our proposed framework complements and extends these directions within the domain of multi-criteria group recommender systems.

5) Clarify the distinction between group shilling and coordinated fake review attacks, as these are conceptually close but differ in structure.

Response: We appreciate the reviewer's insightful comment. To address this suggestion, we have added a clear clarification in the *Related Work* section explaining the conceptual and structural differences between group shilling attacks and coordinated fake review attacks. Specifically, we now emphasize that **group shilling attacks** are rating-based manipulations in which a coordinated group of users injects false profiles or ratings to influence the recommendation generation process (i.e., to push or nuke specific items in the recommendation list). In contrast, **coordinated fake review attacks** primarily target the reputation or perception of items by posting misleading textual reviews rather than numerical ratings. While both involve organized adversarial behavior, the former directly affects the rating matrix and model predictions, whereas the latter operates through opinion spam detection and content-based reputation systems.

6) The explanation of the proposed MC-GSA model is clear, yet the flow could be improved with a diagram legend for Figure 2.

Response: We thank the reviewer for this helpful observation. To enhance the interpretability and visual flow of Figure 2, we have added a diagram legend clarifying the meaning of the symbols and notations used in the framework. Specifically, the legend now defines elements such as u_i (normal users), a_i (attack users), r_{max} (maximum rating), γ_{ij} (filler item ratings), G_n (formed user groups), and the *Top-n List* (recommended items for each group). The figure caption has also been revised to reference this legend. We believe this addition improves readability and helps the reader more easily follow the proposed MC-GSA model.

7) Table 1 mixes notation and textual explanation; please format consistently (e.g., italicize symbols, align descriptions).

Response: We appreciate the reviewer's observation. Table 1 has been carefully reformatted to ensure consistency between the notation and textual descriptions.

8) Algorithm 1 is detailed but lacks complexity analysis or runtime comment—add a brief note on computational cost or scalability.

Response: We appreciate the reviewer's insightful comment. In the revised version, we have added a concise Complexity Note following Algorithm 1 to clarify the computational cost and scalability of the proposed MC-GSA methodology.

9) Tables 3–6 contain rich data but are difficult to read. Summarize key results visually (e.g., line charts or comparative plots for AvgHR and WRV metrics).

Response: We appreciate the reviewer's insightful suggestion to improve the readability of Tables 3–6. In the revised manuscript, we have summarized and visualized the key findings using comparative plots and line charts to complement the tabular data presentation. Specifically, Figures 3 and 4 have been added to illustrate the comparative performance of the two attack models ($GSAGenRan$ and $GSAGenAvg$) across different cluster and filler size settings for both datasets.

10) Add a short paragraph interpreting why certain selection strategies (e.g., MUP-NNZ) cause higher vulnerability—this would enhance insight beyond numerical reporting.

Response: We appreciate the reviewer's valuable suggestion. In the revised manuscript, we have added a new explanatory last paragraph in the Experimental Results section to provide deeper insight into why certain target item selection strategies, such as MUP-NNZ and MUP-Sum, lead to higher vulnerability. Specifically, we explained that these strategies focus on items with low popularity and sparse rating patterns, which makes them less stable in prediction. Consequently, even a small number of injected fake ratings can cause a substantial distortion in their predicted scores.

11) Include a statistical significance statement or standard deviation where applicable to support the reliability of findings.

Response: We appreciate the reviewer's valuable suggestion. In the revised version, a detailed statistical analysis was incorporated to strengthen the reliability of the reported results. Specifically, paired t-tests were conducted to examine the statistical significance of the differences between the $GSAGen_lAvg$ and $GSAGen_lRan$ attack models across different scenarios (S_1 and S_2) and filler

sizes (1% and 5%). The results of these analyses are now presented in Figures 5 and 6, which include p-values and effect size measures (Cohen's d_z) to quantify the magnitude of the observed differences.Furthermore, a detailed interpretation of these results has been added between lines 720–795 in the revised manuscript.

12) Minor grammatical corrections are needed throughout (e.g., "the another study" → "another study"; "criteria-based is intuitively" → "criteria-based scenario is intuitively").

Response: Thank you for your valuable comment and attention. Necessary corrections and errors have been made.

13) Check reference formatting for consistency (e.g., bracketed style [1], [2], [3]) and ensure recent works are included.

Response: We appreciate the reviewer's valuable feedback. The reference section has been thoroughly revised to ensure consistent formatting in bracketed style (e.g., [1], [2], [3]) throughout the manuscript. Additionally, several recent studies (post-2020) have been incorporated into the Related Work section to strengthen the literature coverage and reflect the latest advancements in the field.

14) Figures and tables should include full captions that explain abbreviations (e.g., MUP-DR, AvgHR).

Response: We appreciate the reviewer's observation. To avoid redundancy and improve readability, all abbreviations used in figures and tables are now explained collectively in the methodology section. Therefore, instead of repeating the same explanations in each caption, a consolidated legend and abbreviation list have been provided in the relevant section, ensuring clarity and consistency throughout the manuscript.

Comments by Reviewer #2

The manuscript presents a study on the robustness of multi-criteria group recommender systems against shilling attacks. The research addresses a relevant and timely issue, given the increasing use of group recommendation systems and the potential for malicious manipulation. The study proposes a novel group shilling attack strategy adapted for multi-criteria settings and evaluates its impact. While the study has merit, some aspects of the presentation and interpretation of the results need improvement to ensure accuracy and clarity.

1) Figures and tables should be referenced explicitly in the text, and the key findings from each should be highlighted. It is currently difficult to understand the exact impact of the proposed attack and the comparative performance of the algorithms without carefully scrutinizing the figures.

Response: We sincerely thank the reviewer for this valuable comment. In the revised version, all figures and tables related to the comparative evaluation of attack scenarios have been explicitly referenced and discussed in the text to clarify their implications. Specifically, FigureS 3 and 4 now present the average performance values of the two attack models (*GSAGenRan* and *GSAGenAvg*) across different cluster and filler size settings for both the YM10 and YM20 datasets.

2) The conclusion that multi-criteria systems are "quite robust" needs to be tempered. While the results might indicate a degree of robustness compared to certain benchmarks, it's crucial to avoid overgeneralization. The conclusion should be rewritten to reflect the specific findings of this study, acknowledging the limitations of the attack scenarios and datasets used.

Response: We thank the reviewer for this valuable and constructive comment. In accordance with the suggestion, we have revised the Conclusion section to adopt a more balanced interpretation of the results. —-This study presents a comprehensive analysis of the vulnerabilities of multi-criteria recommender systems to group shilling attacks. The existing literature predominantly focuses on such attacks in single-criteria systems, leaving multi-criteria systems largely unexamined. In this context, our study addresses a critical gap by investigating, modeling, and analyzing shilling attacks specifically in multi-criteria group recommender systems. The proposed attack strategy reveals the susceptibility of multi-criteria recommender systems to contemporary threats. Our findings demonstrate that while these systems offer significant advantages in terms of personalization and detailed evaluations, they can be easily manipulated by malicious user groups. Such manipulations not only decrease the recommendation accuracy but also negatively impact user satisfaction and trust, posing a serious threat to the long-term success and sustainability of recommender systems. The empirical results further show that the impact of attacks increases as the filler size grows. For instance, when the filler size rises from 1% to 5%, the AvgHR values significantly increase, reaching up to 20–25% under average-based attacks. In particular, the MRP aggregation strategy consistently emerged as the most vulnerable, while AU, MUL, and SC were also notably affected. On the other hand, MP and LM demonstrated comparatively greater robustness. These findings emphasize that attack severity is highly dependent on both the attack model and the aggregation technique employed. Also, it is important to note that the robustness observed in this study is specific to the employed datasets and attack models; therefore, the findings should be interpreted within the context of these experimental conditions...—

3) Avoid strong claims about the generalizability of the findings. The study is based on specific datasets and attack models. The conclusions should emphasize that the robustness observed is within the context of these specific experimental conditions.

Response: We appreciate the reviewer's insightful comment. In response, we have carefully revised the Conclusion section to address this concern. Additional statements have been incorporated to clarify that the robustness observed in this study is limited to the specific datasets and attack models used. The revised text also emphasizes that the findings should be interpreted within the context of these experimental conditions, avoiding any overgeneralization.

4) When reviewing your "Related Work" section, I observed that it currently includes only classical shilling attacks (random/average bots), group shilling, and a few referenced studies. While this section provides a good summary of the topic, organizing the literature in a more structured manner or supporting it with a comparative table would make the work stronger and more coherent.

Response: We appreciate the reviewer's valuable suggestion. Accordingly, the Related Work section has been reorganized to present the literature in a more structured manner. In addition, a comparative summary of representative studies has been added as Table 1, which highlights key differences in methodologies, advantages, and contributions of prior works.

5) Since the dataset used in this study is a movie dataset, it would be valuable to note in the Conclusion section that similar analyses could be extended to other domains and datasets in future research to validate the generalizability of the findings.

Response: We appreciate the reviewer's thoughtful comment and agree that emphasizing the potential for broader applicability would strengthen the manuscript. Accordingly, we have revised the Conclusion section to highlight that future research could extend similar analyses to different domains and datasets. This addition aims to acknowledge the limitation of using a single movie dataset and to suggest avenues for validating the generalizability of the proposed findings across

various contexts.
